# First North American case of Hemoglobin Shepherds Bush (β 74[E18] Gly → Asp) in a central Pennsylvania family

**DOI:** 10.1186/1472-6890-14-4

**Published:** 2014-01-15

**Authors:** Scott L Paradise, Lauren Estep, Jordan Olson, Keri Donaldson

**Affiliations:** 1 Department of Pathology and Laboratory Medicine, Penn State Hershey Medical Center, Hershey, PA, USA

**Keywords:** Hemoglobin, Shepherds bush, Hemolytic anemia

## Abstract

**Background:**

Hemoglobin Shepherds Bush (Human Genome Variation Society name: HBB:c.224G > A) is an unstable hemoglobin variant resulting from a β 74 GGC to GAC mutation (Gly to Asp) that manifests clinically as hemolytic anemia or gall bladder disease due to chronic subclinical hemolysis.

**Case presentation:**

We report a Pennsylvania family of English descent with this condition, first noticed in a 6-year-old female. The proband presented with splenomegaly, fatigue, dark urine and an elevated indirect bilirubin. Hemoglobin identification studies and subsequent genetic testing performed according to a systematic algorithm elucidated the diagnosis of Hb Shepherds Bush.

**Conclusions:**

This is the first case of this rare hemoglobin variant identified in North America to our knowledge. It was identified using a systematic algorithm of diagnostic tests that should be followed whenever considering a rare hemoglobinopathy as part of the differential diagnosis.

## Background

Hemoglobin (Hb) Shepherds Bush (β 74[E18] Gly → Asp, Human Genome Variation Society name: HBB:c.224G > A) is an unstable hemoglobin molecule resulting from a β 74 GCC to GAC point mutation (Gly to Asp). The lesion is in the β chain, so HbA_2_ and HbF molecules are unaffected although they may exist in increased amounts. The resultant hemoglobin molecule has increased oxygen affinity and a decreased response to 2,3-DPG [1].

Because of its instability, patients with Hb Shepherds Bush may experience acute hemolytic episodes, secondary to inciting illnesses or drugs [2], and subclinical hemolysis may occur between acute hemolytic episodes. Subclinically, hemolysis may manifest as gallbladder disease from calcium bilirubinate bile pigment stones [2-5]. The hemolysis can often be characterized by Heinz bodies on a Heinz preparation in which precipitated hemoglobin binds to the red cell membrane decreasing the red cell’s deformability and causing the red cell’s premature destruction in the spleen, bite cell creation and basophilic stippling [6]. Splenomegaly may occur due to the elevated intravascular hemolysis with patients usually improving post-splenectomy. Patients in the heterozygous state usually present with symptoms that are similar but less severe compared to patients in the homozygous state.

Two cases of heterozygous Hb Shepherds Bush in the literature demonstrated improvement in red cell survival from 10 and 11 to 18 and 20 days, respectively after splenectomy [4]. Of note, the seventeen reported patients with this particular hemoglobinopathy, apart from one South African female of English extraction, [2] have all been of Spanish [7] and Sicilian [3,4,8] heritage; none of whom lived in North America. Our patient is the first identified case of Hb Shepherds Bush in a North American family making her, and the rest of her family, unique.

## Case presentation

A 6-year-old Caucasian female from central Pennsylvania with a self-reported English heritage presented to her primary care physician complaining of fatigue, icterus, dark urine and intermittent abdominal pain associated with nausea and vomiting. Physical exam showed a normally developing child whose weight was 24.1 kg, height 113.5 cm, temperature 36°C, blood pressure 123/68 mmHg and pulse 109. She had mild abdominal tenderness and splenomegaly. The patient’s past medical history was negative for any major diseases, her immunizations were up to date and her birth history was normal with no record of neonatal jaundice. Her father and paternal uncle were known to have anemia, and her paternal grandmother and aunt both had splenectomies when they were young. The patient was referred to a pediatric hematologist for further examination.

**Figure 1:**
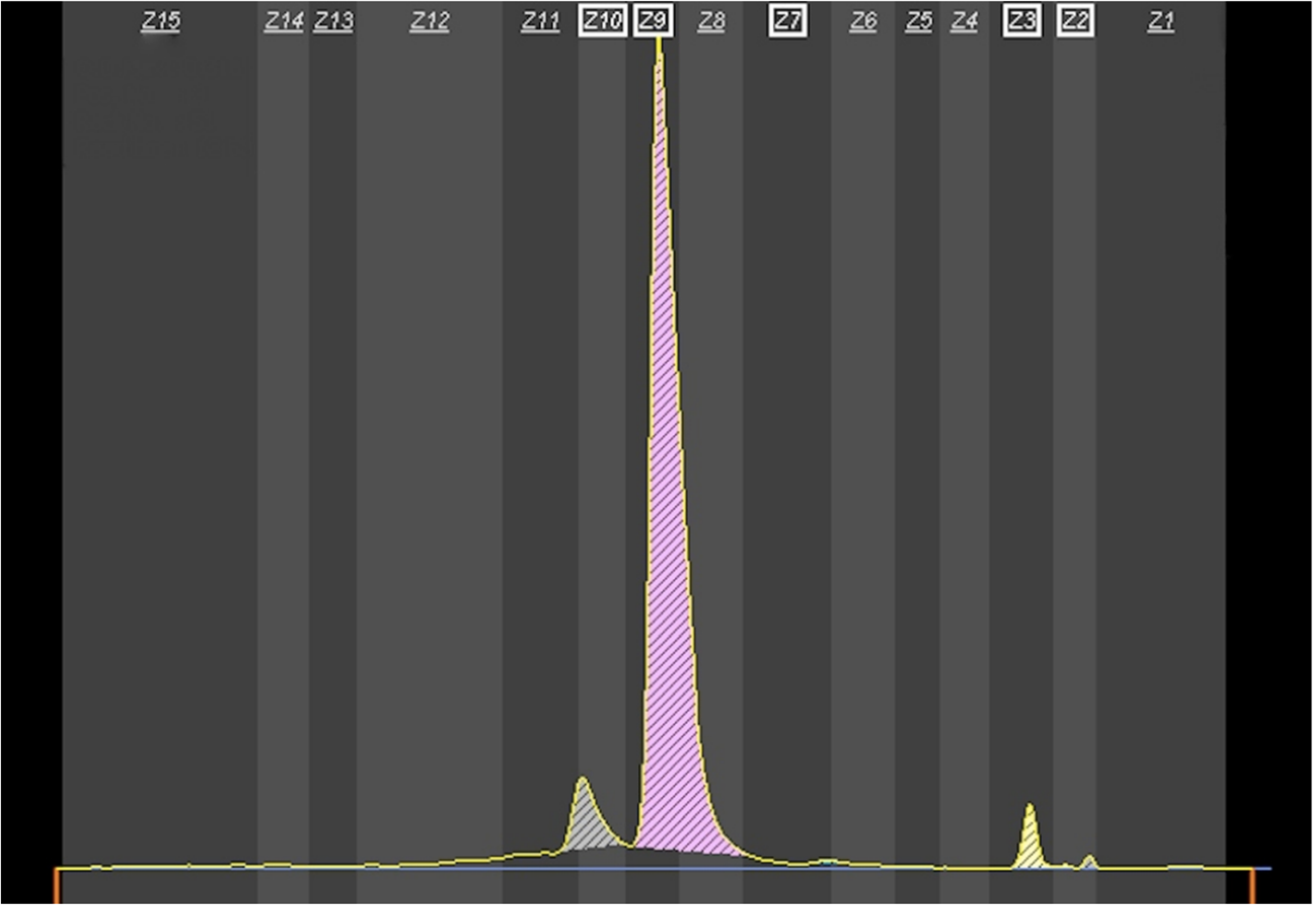
**Capillary zone electrophoresis of the patient’s hemoglobin.** CE shows the hemoglobin variant eluting with normal hemoglobins. Note peaks corresponding to Hb Shepherds Bush (6.5% of total, zone 10), HbA (89.6%, zone 9), HbF (0.3%, zone 7), HbA2 (3.2%, zone 3) and hemoglobin breakdown products (0.4%, zone 2). This chromatogram was obtained after a transfusion following a parvovirus infection described later accounting for the small amount of her native hemoglobin compared to HbA.

Laboratory testing ordered by her pediatric hematologist demonstrated anemia (Hgb 10.9 g/dL), mild macrocytosis (94.3 fL) and significant reticulocytosis (391,200 cells/μL or 10.16%). Capillary zone electrophoresis (CE) demonstrated migration of several peaks: one of which was an unknown hemoglobin variant (image not available). A CE chromatogram performed at a later date after transfusion (Figure 1) is included to show the migration pattern of our patient’s hemoglobin variant, which eluted slightly earlier than HbA.

**Figure 2:**
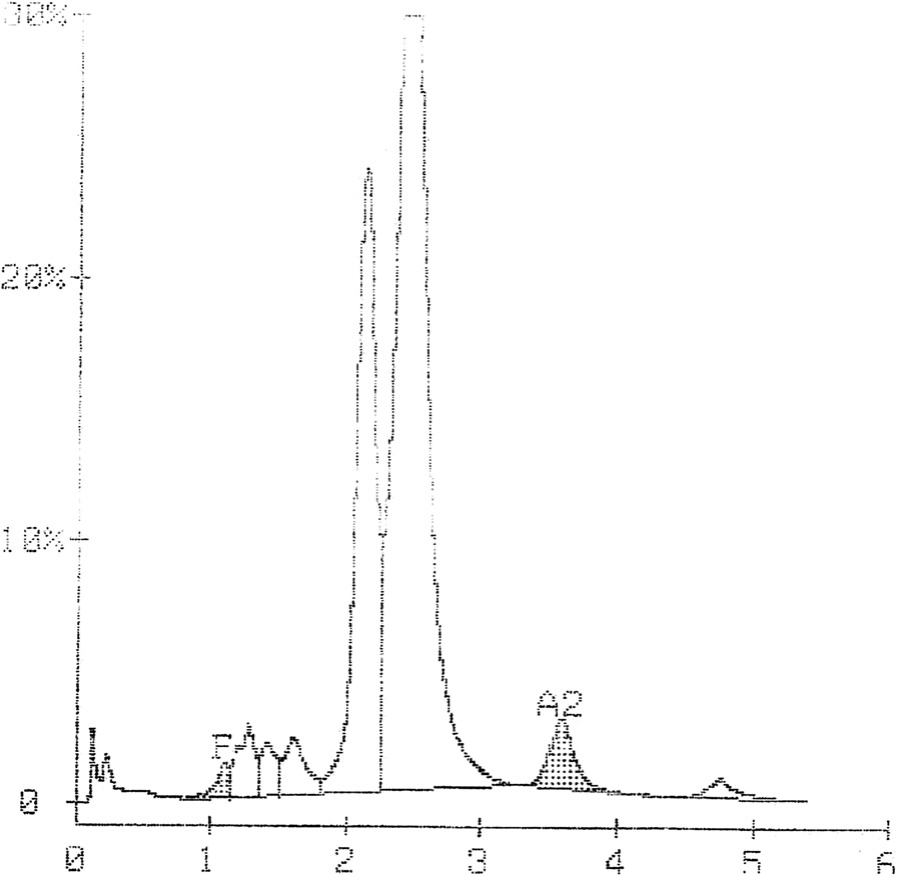
**High performance liquid chromatography analysis of the patient’s hemoglobin.** HPLC analysis shows a peak of unknown variety at 2.13 min that eluted earlier than HbA at 2.44 min. The chromatogram also contains peaks corresponding to HbF and HbA_2_.

**Figure 3:**
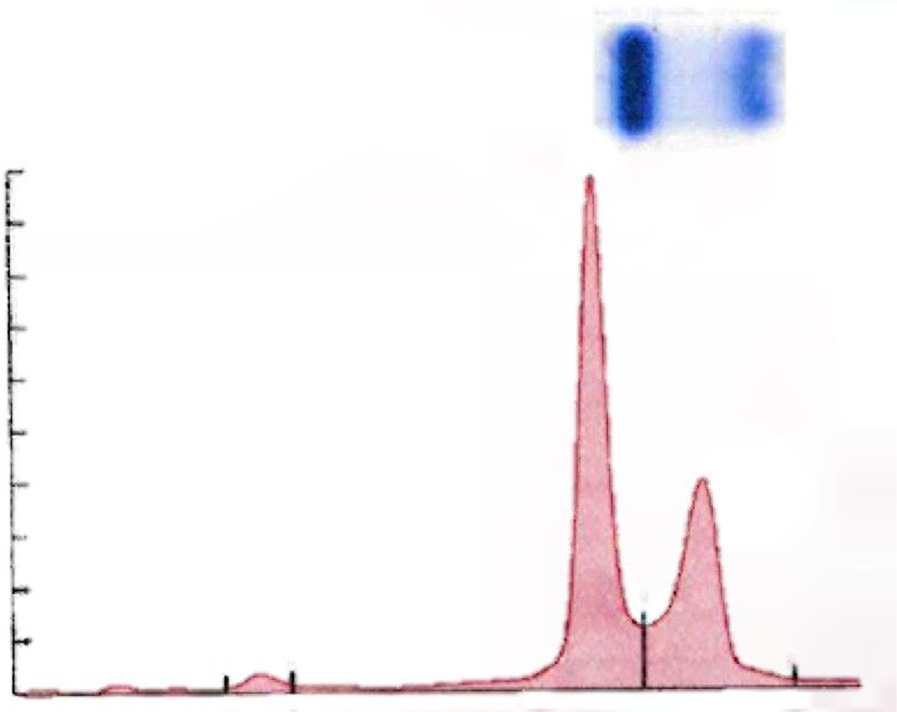
**Alkaline hemoglobin electrophoresis of the patient’s hemoglobin.** Alkaline hemoglobin electrophoresis on cellulose acetate with densitometer tracing superimposed below electrophoresis bands shows a dense band where HbA is expected to appear and a second band that has migrated more anodally indicating an unknown hemoglobin variant.

Our patient’s blood sample was then sent to a national reference laboratory for further characterization. Using high performance liquid chromatography (HPLC) for hemoglobin (Figure 2), several hemoglobin types were demonstrated. One of the hemoglobin forms, and unknown variant (34.6% of total), eluted slightly earlier than HbA (61.2%). Alkaline gel electrophoresis was also utilized to demonstrate the migration of the unknown hemoglobin variant, which migrated slightly more anodally than HbA (Figure 3).

With an unknown variant present, polymerase chain reaction (PCR) sequence analysis of the patient’s hemoglobin genes was performed at the same national reference laboratory, which determined that this unknown variant was Hemoglobin Shepherds Bush. Once diagnosed, our patient’s father and paternal uncle tested positive for the same mutation.

After diagnosis, our patient was vaccinated against encapsulated bacteria and followed yearly to monitor her splenomegaly. At age 9, she presented to her pediatrician with a 5-day course of upper respiratory symptoms and fever. She was tachycardic, and laboratory tests revealed anemia (Hgb 6.0 g/dL), a reticulocyte percentage of 0.26% and a low platelet count (110,000 /μL). Because of these abnormal findings, she was admitted to a pediatric hematologist who found her to be positive for IgG and IgM titers for anti-parvovirus antibody. This acute infection accounted for the patient’s upper respiratory symptoms, fever and anemia. She was transfused with 1 unit of packed red blood cells to correct her anemia and was discharged after clinical symptom improvement.

At age 10, she presented to us with visible jaundice and an enlarging spleen demonstrated on ultrasound. Although no history of gallstones, she appeared to be at significant risk for cholelithiasis based on her chronic hemolytic anemia caused by Hb Shepherds Bush. Our patient was therefore treated with laparoscopic splenectomy and cholecystectomy. Pathologic review of her gallbladder revealed evidence of chronic cholecystitis. After uneventful recovery from surgery, she was discharged home and continues to do well.

## Conclusions

Hb Shepherds Bush (HBB:c.224G > A) is an unstable hemoglobin molecule resulting from a β 74 GCC to GAC point mutation (Gly to Asp). Hb Shepherds Bush, although initially reported in a South African woman of English extraction [2], has since been found in patients of only Sicilian [3,4,8] or Spanish [7] decent. This case thus illustrates the potentially variable genetic diversity of the condition. Our patient’s family identifies itself as of English heritage, however there is no direct evidence of a common English ancestry link with the first reported case of Hb Shepherds Bush [2]. Her case, however, is the first recorded in North America where her family has resided for several generations making her unique.

When patients present with the symptoms of an acute hemolytic episode—fatigue, hepatosplenomegaly, dark urine, scleral icterus and abdominal pain [9]—a systematic algorithm should be utilized to determine the cause of their presentation. In the setting of acute anemia—demonstrated on CBC and reticulocyte percentage—hemoglobinopathies should be considered only after ruling out treatable causes such as drugs, infection (via negative cultures) or autoantibodies (via a negative Direct Coombs test) which can also produce the aforementioned symptoms. If the above are ruled out—and laboratory tests indicate anemia, reticulocytosis and signs of active hemolysis—then the physician should begin to consider a congenital hemoglobinopathy as a possible diagnosis.

If abnormal hemoglobin is suspected, then a battery of tests should be used to determine the specific hemoglobin variant. Alkaline hemoglobin electrophoresis on cellulose acetate is commonly used to identify major hemoglobin variants with acidic pH electrophoresis on citrate agar often utilized as an auxiliary step to distinguish among hemoglobin variants that co-migrate under alkaline conditions. If higher resolution is needed when using electrophoresis, methods such as isoelectric focusing can be explored. In our patient’s case, isoelectric focusing was not necessary to distinguish the presence of an abnormal hemoglobin. Under alkaline conditions, Hb Shepherds Bush separates from HbA and migrates in a manner similar to HbJ.

If electrophoresis is positive for an unknown hemoglobin variant, then HPLC (both cation exchange and reverse phase) should be utilized to evaluate rare hemoglobinopathies. In addition to chronicling elution times for identification of the hemoglobin variant, HPLC also provides percent compositions of blood samples. CE can also provide a quantitative evaluation of hemoglobin types [10,11].

In the evaluation of our patient, CE was performed initially per our institution’s standard operating procedures as an automated and high-throughput screening tool. After positive results were obtained using CE, the patient’s blood sample was sent to a reference laboratory for further analysis where alkaline gel electrophoresis was performed along with HPLC, each showing migration of an unknown hemoglobin variant.

If HPLC or CE is positive for an unknown hemoglobin variant, PCR, which can be performed on genomic DNA extracted from a peripheral blood sample, should be used to determine genetically the hemoglobinopathy of study [12-14]. PCR sequencing can be utilized to find the abnormal mutations that generate the variant hemoglobin causing the hemoglobinopathy. In our patient, PCR sequencing showed the β 74 GCC to GAC point mutation (Gly to Asp) mutation specific to Hb Shepherds Bush.

When our patient’s specific hemoglobinopathy was elucidated, the patient was treated symptomatically with packed red blood cell transfusions. Long-term treatment plans should avoid iron supplementation and frequent transfusions as to prevent iron overload. Patients should also be watched carefully for gall bladder disease caused by calcium bilirubinate precipitating as pigmented bile stones [5].

Our patient’s case demonstrates a classic finding of hemolytic anemia caused by an unstable hemoglobin variant. Although Hb Shepherds Bush is uncommon, the diagnosis of this disease illustrates progression through a systematic algorithm of tests to consider when dealing with possible congenital hemoglobinopathies of unknown characterization. The first reported case of Hb Shepherds Bush in a North American family and in a patient of non-Sicilian, non-Spanish descent, this case illustrates an increased geographic and genetic diversity of the disease of Hb Shepherds Bush, and it demonstrates a diagnostic approach to unknown hemoglobinopathies.

### Consent

Written informed consent was obtained from the patient and patient’s legal guardian for publication of this Case report and any accompanying images. A copy of the written consent is available for review by the Editor of this journal.

## Abbreviations

Hb: Hemoglobin; CE: Capillary zone electrophoresis; HPLC: High performance liquid chromatography; PCR: Polymerase chain reaction.

## Competing interests

The authors declare that they have no competing interests.

## Authors’ contributions

SLP helped to draft the manuscript. LE helped to draft the manuscript. JO conceived of the study and reviewed the manuscript. KD revised the manuscript. All authors read and approved the final manuscript.

## Pre-publication history

The pre-publication history for this paper can be accessed here:

http://www.biomedcentral.com/1472-6890/14/4/prepub

## References

[B1] May A Huehns ER The control of oxygen affinity of red cells with Hb-Shepherds Bush Br J Haematol 1972 22 599 607 10.1111/j.1365-2141.1972.tb05706.x 5032098

[B2] White JM Brain MC Lorkin PA Lehmann H Smith M Mild “unstable haemoglobin haemolytic anaemia” caused by Haemoglobin Shepherds Bush (B74 (E18) Gly → Asp) Nature 1970 225 939 941 10.1038/225939a0 5415129

[B3] Sansone G Sciarratta GV Genova R Darbre PD Lehmann H Haemoglobin Shepherds Bush (beta 74 [E 18] Gly leads to Asp) in an Italian family Acta Haematol 1977 57 102 108 10.1159/000207866 402764

[B4] Schiliro G Musumeci S Ruso G Marinucci M Giampaolo A HB Shepherds Bush in two Italian carriers Hemoglobin 1981 5 493 496 7275665

[B5] Yates AM Mortier NA Hyde KS Hankins JS Ware RE The diagnostic dilemma of congenital unstable hemoglobinopathies Pediatr Blood Cancer 2010 55 1393 1395 10.1002/pbc.22702 20730880 PMC4347403

[B6] McPherson RA Pincus MR Henry’s Clinical Diagnosis and Management by Laboratory Methods 2011 Philadelphia: Saunders

[B7] Carreño DL Villegas A Sanchez-Varela JM HB shepherds bush [β74 (E18) GLY → ASP]: the first case in a Spanish woman Hemoglobin 1993 17 243 246 10.3109/03630269308998898 8330976

[B8] Dibenedetto SP Samperi P Tumminelli G Testa R Gagliano M Mancuso M Hemoglobin Shepherds Bush in a Sicilian family Minerva Pediatr 1990 42 193 196 2381392

[B9] Simon TL Snyder EL Stowell CP Strauss RG Solheim BG Petrides M Rossi's Principles of Transfusion Medicine 2011 Bethesda: Wiley-Blackwell

[B10] Chen FT Liu CM Hsieh YZ Sternberg JC Capillary electrophoresis—a new clinical tool Clin Chem 1991 37 14 19 1988203

[B11] Borbely N Phelan L Szydlo R Bain B Capillary zone electrophoresis for haemoglobinopathy diagnosis J Clin Pathol 2013 66 29 39 10.1136/jclinpath-2012-200946 23105123

[B12] Smetanina NS Molchanova TP Huisman TH Analysis of mRNA from red cells of patients with thalassemia and hemoglobin variants Hemoglobin 1997 21 437 467 10.3109/03630269708993129 9322078

[B13] Clark BE Thein SL Molecular diagnosis of haemoglobin disorders Clin Lab Haematol 2004 26 159 176 10.1111/j.1365-2257.2004.00607.x 15163314

[B14] Coleman WB Tsongalis GJ Molecular diagnostics: for the clinical laboratorian 2006 Totowa: Human Press

